# Activation-Inhibition dynamics of the oscillatory bursts of the human EEG during resting state. The macroscopic temporal range of few seconds

**DOI:** 10.1007/s11571-021-09742-6

**Published:** 2021-11-07

**Authors:** Carlos M. Gómez, Brenda Y. Angulo-Ruíz, Vanesa Muñoz, Elena I. Rodriguez-Martínez

**Affiliations:** 1 Human Psychobiology lab, Experimental Psychology Department, Psychology school, University of Sevilla, c/Camilo José Cela s/n, 41018 Sevilla, Spain; 2 Evolutive and Developmental Psychology Department, Psychology school, University of Sevilla, Sevilla, Spain

**Keywords:** Oscillatory network activity, Oscillatory bursts, Amplitude duration relationships, Activation-inhibition dynamics, Frequency content, Resting-state EEG

## Abstract

The ubiquitous brain oscillations occur in bursts of oscillatory activity. The present report tries to define the statistical characteristics of electroencephalographical (EEG) bursts of oscillatory activity during resting state in humans to define (i) the statistical properties of amplitude and duration of oscillatory bursts, (ii) its possible correlation, (iii) its frequency content, and (iv) the presence or not of a fixed threshold to trigger an oscillatory burst. The open eyes EEG recordings of five subjects with no artifacts were selected from a sample of 40 subjects. The recordings were filtered in frequency ranges of 2 Hz wide from 1 to 99 Hz. The analytic Hilbert transform was computed to obtain the amplitude envelopes of oscillatory bursts. The criteria of thresholding and a minimum of three cycles to define an oscillatory burst were imposed. Amplitude and duration parameters were extracted and they showed durations between hundreds of milliseconds and a few seconds, and peak amplitudes showed a unimodal distribution. Both parameters were positively correlated and the oscillatory burst durations were explained by a linear model with the terms peak amplitude and peak amplitude of amplitude envelope time derivative. The frequency content of the amplitude envelope was contained in the 0–2 Hz range. The results suggest the presence of amplitude modulated continuous oscillations in the human EEG during the resting conditions in a broad frequency range, with durations in the range of few seconds and modulated positively by amplitude and negatively by the time derivative of the amplitude envelope suggesting activation-inhibition dynamics. This macroscopic oscillatory network behavior is less pronounced in the low-frequency range (1–3 Hz).

## Introduction

The presence of oscillations is ubiquitous along the nervous system, from very low frequencies to very high-frequency (Niedermeyer and Lopes da Silva 1999). The neurophysiological characteristics of brain oscillations of EEG resting state have been classically defined by the spectral power, which indicates the energy included in a certain frequency range, topography, and psychophysiological reactivity (Valdés-Sosa et al., 1990). However, brain oscillations occur in transient bursts of activity. It has been proposed that these oscillation bursts are the primary ingredients of oscillatory activity that can be observed over the scalp (Feingold et al. 2015; Shin et al. 2017). The oscillatory bursts have been recently characterized as wave packets (Pal and Panigrahi 2020). The common procedure of averaging power would be obscuring the natural neurodynamics of oscillatory bursts. These bursts of oscillatory activity would be the background that determines cognitive and behavioral activity (Neymotin et al. 2020; Lakatos et al. 2019). Indeed, the Entrainment Theory suggests that attention is highly dependent on the phase resetting of oscillations, permitting a network optimal response (Lakatos et al. 2008,2019). Related to this concept, the Communication Through Coherence Theory highlights the phase coherence of oscillations in different networks to permit coordinated transmission and processing of information in functionally related areas (Fries 2015).

Different criteria have been proposed to characterize burst oscillations. The two main criteria are related to defining (i) a burst as a power peak in frequency and time that exceeds a threshold (Sherman et al. 2016), an alternative view (ii) is to consider an amplitude peak of a given oscillatory frequency in which the next local peak is separated by a few complete cycles of these oscillations (Lundqvist et al. 2016); or a combination of both criteria, as we would follow in the present report. The duration criterion is important to differentiate a true oscillation from a transient event, for instance, an evoked potential. The presence of oscillations would be related to the recurrent activity of neural circuits that would produce cycles of extracellular potentials in a given frequency (Wang 2010).

This new conceptualization of oscillatory bursts of neural activity is suggesting burst rate as a new parameter to describe the oscillatory activity (van Ede et al. 2018). The measurement of parameters derived from describing brain activity as a succession of bursts requires to identify what characteristics must have a burst to be considered a genuine oscillatory burst. The method most used is to detect an amplitude threshold in the envelope of the oscillatory activity, however, different thresholds have been used as 2 standard deviations on the mean (Lundqvist et al. 2016); 6 times the median (Shin et al. 2017); and the 98th percentile (Sherman et al. 2016). And in general, there is not a particular criterion to define the threshold level and it must follow a data-driven approach, although certainly, a higher threshold would identify a lower number of individual oscillatory events than low amplitude thresholds.

Furthermore, it has been suggested that it is necessary to include a few cycles of oscillatory activity to consider a given oscillatory burst as a true oscillation and not a transient response. (Hughes et al. 2012). More sophisticated models to extract the presence of an oscillation burst based on a hidden Markov model have shown a great coincidence with the thresholding approach (Quinn et al. 2019; Seedat et al. 2020); and an approach based on cycle-by-cycle approach have also been proposed (Voytek et al., 2019). These latter two approaches emphasize the need of determining periods in which oscillations are present concerning those in which are not present and are an artifact of the filtering and Fourier procedures to allocate energy in each frequency at any time. In the same vein, it has been suggested that methods derived from Fourier and Hilbert transform are not appropriate for analyzing oscillatory bursts because they always would assign a certain amplitude to oscillations at any time (Bartz et al. 2019; Cole and Voytek 2019). However, the view of low amplitude constant oscillatory activity with the presence of some high amplitude short-lived bursts of oscillatory activity can be obtained with Fourier methods, and then be reconciled with methods suited for extracting high amplitude short-lived oscillatory bursts obtained by stringent thresholding. As it can be deduced from the present paragraph the methodological approaches to define the extent and parameters to define a burst of brain oscillations are diverse, although all these differences approaches suggest the need for a characterization of oscillatory activities at different frequencies to correctly characterize brain dynamics. In the same vein, the approach followed in the present report is more related to an energetic approach trying to explain the temporal distribution of energy of oscillatory bursts, as obtained from Fourier and Hilbert methods, than extracting conspicuous oscillatory bursts from the background EEG. The latter having a higher potential for transmitting information across the brain.

It has been proposed that bursts of oscillatory activity can be accounted by four different scenarios, (i) sustained rhythmic activity, (ii) amplitude modulated rhythmic activity due to instantaneous inputs and excitation-inhibition balance, (iii) oscillatory bursts generated in a rather stochastic pattern without underlying continuous rhythmic activity, and (iv) a certain amplitude threshold must be crossed in the underlying rhythmic activity to produce a measurable burst (van Ede et al. 2018). A recent interesting result (Neymotin et al. 2020) suggests, by computing the squared coefficient of variation and fano factor of the number of oscillatory events in predefined time windows of certain durations in a resting state condition, that the presence of oscillatory bursts occur in a rather rhythmic pattern.

The amplitude modulated continuous oscillatory activity is considered as a standard model by van Ede et al. (2018), while the presence of a threshold would implicate some type of cooperative phenomena with a certain resemblance to the action potentials in terms of activation-deactivation when compared to the process of depolarization-repolarization of the action potential. An alternative view is the presence of clearly defined short-lived oscillatory burst (Sherman et al. 2016). Anyway, the selection of a threshold in amplitude or number of cycles imposes a dependence on the chosen criteria to define the limits of an oscillatory burst. Therefore, the dependence of any extracted parameter obtained to quantify bursts oscillations must take into account the constraints imposed by the methodological criteria selected to extract bursts oscillations from background EEG. An approach searching for oscillations of high amplitude, and not considering oscillatory activity as a continuous would not explain the total energy included in the EEG, which is the approach followed in the present report. Both approaches are complementary, (i) the method followed here permits to consider all oscillatory bursts present in the EEG and explains the whole energy of the EEG spectrum, and (ii) methods that would select the more conspicuous oscillatory bursts which would be those with higher impact in brain information processing.

The objective of the present report is to define the statistical characteristics of duration and peak amplitudes of bursts oscillations during resting state in humans. The present approach would consider a minimum number of cycles and amplitude to consider an oscillation burst as independent from others. But no single period would be considered as free of oscillatory activity (Shin et al. 2017; van Ede et al. 2018; Sherman et al. 2016) and a burst would start and would end as soon as a change in the amplitude trend is detected. The reconstruction of the non-filtered signal from the filtered signals would permit to give consistency to the presence of true oscillations, no matter if they have periods of low amplitude. As the present report is exploratory no previous hypothesis can be made about the possible duration of oscillatory bursts at different frequencies, although a positive relationship is expected between the duration and the amplitude of the oscillatory bursts.

The construction of histograms of peak amplitude would permit to observe if bimodal distributions, compatible with a thresholding mechanism, or unimodal more compatible with a continuous modulation of oscillatory burst amplitudes are present. The Power Spectral Density (PSD) of the envelope of oscillations would permit to define the frequency content of the amplitude modulation of brain oscillations forming oscillatory bursts. The present macroscopic approach must be considered complementary of microscopic approaches using intracerebral electrodes and would reveal oscillatory brain dynamics at a macroscopic level. Therefore, the specific objectives of the present report are, (i) to describe the statistical properties of oscillatory burst durations and amplitudes in a broad frequency range (1–99 Hz), and (ii) to analyze the possible interdependence between these two parameters.

## Methods

### Participants

A sample of 5 subjects (1 male of 18 years, and 4 females of 18,19,22, and 25 years) was selected from a recorded population of 40 adult subjects. The criterion for inclusion in the present analysis was to have no rejection of any period in the EEG due to artifacts. This inclusion criterion was imposed to permit to have an EEG recording of 180 s with no artifact contamination. With a continuous recording without artifacts, it would be possible to estimate the duration of a high number of burst oscillations, including those of very low frequencies that necessarily would have long durations. Experiments were conducted with the informed and written consent of each participant, following the Helsinki Protocol. The study was approved by the Bioethical Committee of the Junta de Andalucía.

### Experimental session

The EEG resting-state activity, also termed as non-task-related activity, was recorded for 3 min in open eyes condition. The subjects were asked to blink as little as possible while looking at the screen. Thirty-two electrodes installed on an electrode cap (ELECTROCAP) permitted to register the EEG employing an analog–digital acquisition system (ANT amplifiers, The Netherlands) (electrodes: Fp1, Fpz, Fp2, F7, F3, Fz, F4, F8, FC5, FC1, FC2, FC6, M1, T7, C3, Cz, C4, T8, M2, CP5, CP1, CP2, CP6, P7, P3, Pz, P4, P8, POz, O1, Oz, O2). Two electrodes situated on the outer edge of each eye and two electrodes located above and below the left eye permitted to record the horizontal and vertical eye movements, respectively. The recordings were made with an average reference and were re-referenced offline to the average mastoid (M1 + M2)/2. Impedance was maintained below 10 kilo-ohms during the EEG recording. Amplification gain was 20 K. The recordings were made in direct current (DC) mode at 512 Hz as the sampling rate.

### Data analysis preprocessing

**Fig. 1:**
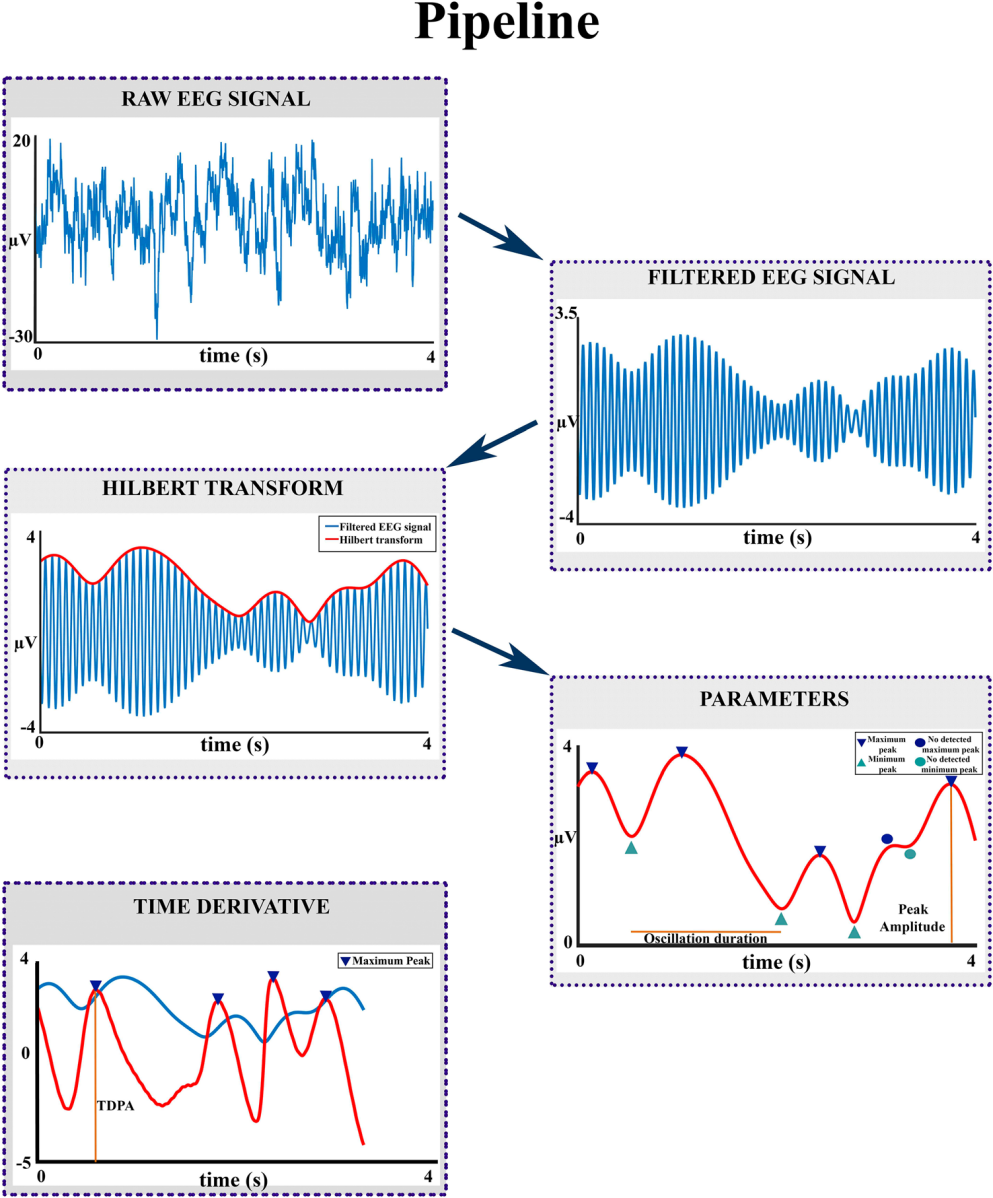
Processing pipeline. The raw EEG was filtered in different frequency ranges of 2 Hz, from 1–3 Hz to 97–-99 Hz. The envelope of the filtered signal was obtained employing the analytic Hilbert transform. The time derivative of the amplitude envelope obtained with the Hilbert transform was also obtained. Peaks and troughs of the envelope were found (see rectangles). The circles represent a peak and through that were not extracted because of low prominence. Finally, the parameters of amplitude and duration of the amplitude envelope and the peak amplitude of the time derivative of the amplitude envelope (TDPA) were obtained for each oscillatory burst at any frequency range and electrode

EEG recordings were preprocessed using the EEGLAB software package (Delorme & Makeig 2004). Different types of artifacts (eye movements, blinks, muscle artifacts, and the interference from the alternating current electric line contributions) were eliminated by identifying and removing the independent components (ICAs) related to those artifacts and then reconstructing the EEG signals. Two-second epochs were created and those in which the EEG exceeded ± 120 μV on any channel were discarded. As indicated before only 5 of the 40 recorded subjects presented all trials free of artifacts. This protocol permitted to keep 5 subjects in which no trials were rejected over the 90 recorded trials. Then the trials of these 5 subjects were concatenated to have a continuous recording of 180 s. The pipeline analysis displayed in Fig. 1 was applied to the 180 s concatenated EEG for all the analyzed frequencies. The raw EEG data of these five subjects can be found in https://github.com/breangrui/raw-data-EEG-oscillations.

### Extraction and analysis of oscillatory bursts

**Fig. 2:**
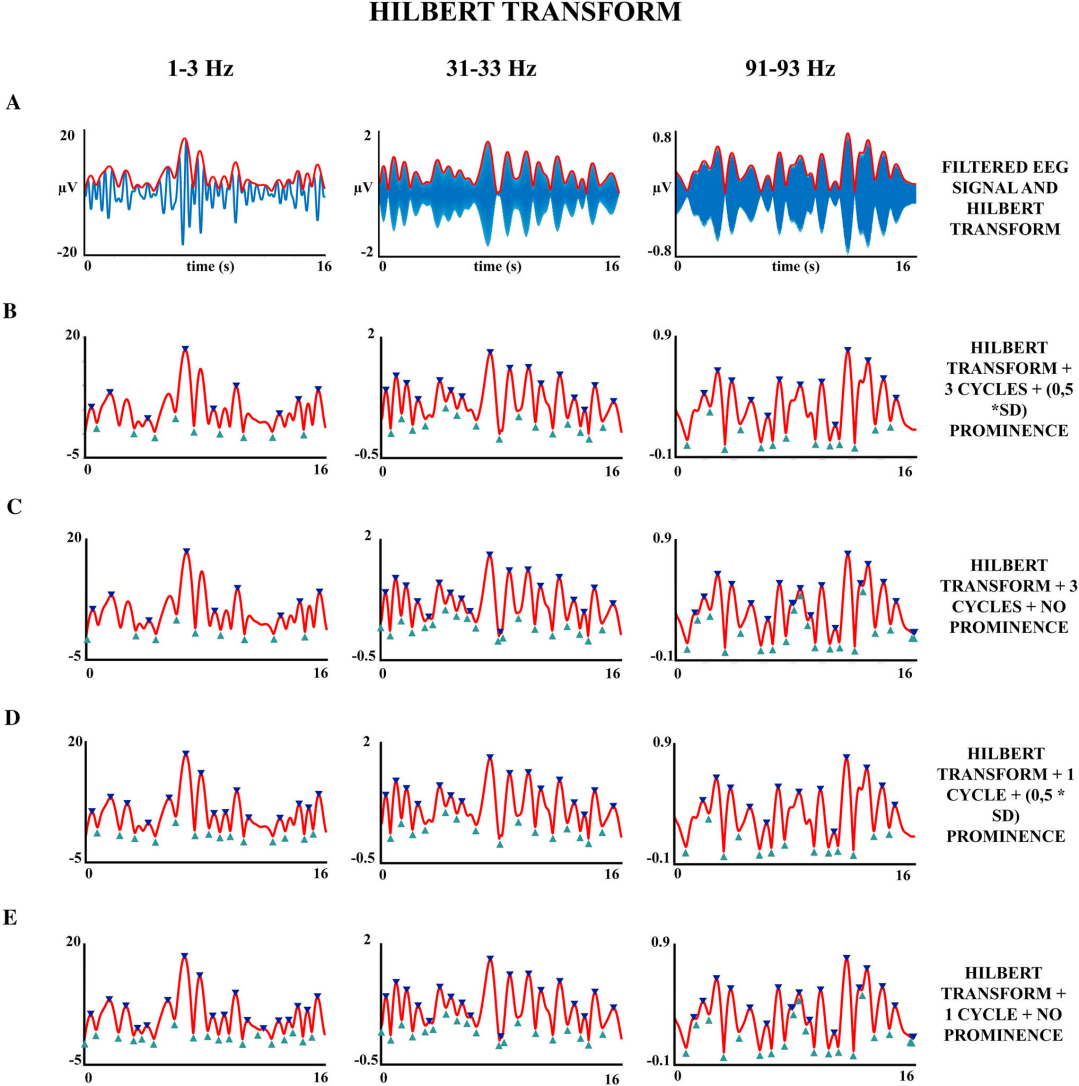
Effect of the number of cycles and prominence parameters for defining the extent of oscillatory bursts. The location of peaks and troughs of the envelope of the filtered signal was computed varying the parameters number of cycles and prominence. Arrows indicate the temporal limits of the extracted oscillatory bursts. **a** Filtered oscillations and envelope of oscillatory bursts obtained by the Hilbert transform. **b** Oscillatory bursts obtained applying a minimum of 3 cycles and prominence of 0.5 *SD as selection parameters. **c** Applying 3 cycles and no prominence as parameters. **d** 1 cycle and 0.5*SD prominence as parameters. **e** Applying 1 cycle and no prominence

The characterization and analysis of oscillatory bursts were computed using fieldtrip (Oostenveld et al. 2011) and Matlab scripts (Matlab R2020b). The present approach to extract oscillations was based on the hypothesis that oscillations were continuously present in the EEG although at very different amplitudes, which corresponds to the hypothesis (ii) described in the introduction section (van Ede et al. 2018). This approach can be physiologically valid taking into account that extracranial electrodes record brain areas of a few squared centimeters and then the EEG signal corresponds to a multinetwork macroscopic signal. This macroscopic approach cannot discard the possibility that at the microscopic level oscillatory bursts would have a lower duration than macroscopic bursts oscillation. However, if applying the continuous oscillation model at a macroscopic level, a relatively organized oscillatory pattern is obtained in the EEG, then the validity of the presence of oscillatory bursts at macroscopic levels would be substantiated. As an initial approach to validate the presence of continuous macroscopic oscillatory activity in the EEG, the band-pass filtered EEG would be inspected (Figs. 1 and 2).

The following pipeline (Fig. 1) was used to analyze the data. Data were bandpass filtered in 32 steps of 2 Hz wide bandpass frequency ranges (1–3 Hz, 4–6 Hz, 7–9 Hz………91–93 Hz, 94–96 Hz, and 97–99 Hz; the 49–51 Hz range was not analyzed to avoid AC line artifacts). The fieldtrip function *ft_preproc_bandpassfilter using* an FIR filter (Matlab *firl* function), zero-phase forward, and reverse filter was used using a 3000th order. Then the analytic Hilbert transform was applied to the filtered data to obtain the phase and the envelope amplitude of the filtered data, using the fieldtrip function *ft_preproc_hilbert.* The time derivative of the amplitude envelope was computed applying the *diff* function of Matlab using a time interval of 2 ms. In fact, this function computes the derivative by subtracting two adjacent recorded points and dividing by the time interval between them.

Peaks and troughs of the amplitude envelope were obtained, imposing two conditions: (i) a minimum number of three cycles for an oscillatory burst to be considered an independent burst; and (ii) an amplitude prominence of the peak amplitude of the envelope higher than 0.5*SD (SD: Standard deviation of the envelope of the analyzed frequency). The prominence is a parameter that provides an amplitude measurement relative to the adjacent peaks. The use of prominence permits to eliminate fluctuations lower than 0.5*SD in amplitude relative to the encircling peaks, and not as an absolute measurement of the peak amplitude. For instance, the points labeled by circles in Fig. 1 would not be considered as an independent oscillatory burst because of the small prominence with the peak situated at its left. Normalizing the threshold by the SD of each frequency and subject permits us to take into account that low-frequency oscillations present higher amplitude than higher frequencies, but also the possible differences in amplitude in different subjects. To validate that the chosen parameters of a minimum of 3 cycles and prominence of 0.5*SD are good parameters to extract oscillations bursts, other thresholding and number of cycles parameters were visually inspected. Figure 2 shows a comparison of the oscillation bursts extracted with 0.5*SD prominence and no prominence, and 3 cycles and no cycles, as threshold parameters to be considered as an oscillatory burst. Although the procedure is somehow qualitative the parameters of 3 cycles and 0.5*SD prominence were accepted as optimal to continue the extraction and analysis of oscillatory bursts. The selection of three cycles was imposed to deal with true oscillations and not with phasic responses in the bandpass filtered frequencies.

The following parameters from the oscillations bursts were obtained for each range of frequency, electrode, and subject: duration and peak amplitude of the amplitude envelope of oscillatory bursts, and the peak of the amplitude envelope time derivative (Fig. 1). The frequency histograms of the oscillatory bursts duration and peak amplitudes of the envelope were computed for each subject, electrode, and frequency range. The mode of the histograms of burst oscillatory duration and amplitude were computed and represented as histograms of the modes. The topography of the oscillatory bursts for all the analyzed frequency ranges was obtained by displaying the mean value of the oscillations envelope across the 180 ms recorded in each electrode, using the *topoplot* function of *EEGLAB*.

The Spearman correlation of amplitude vs. duration of the oscillatory bursts was computed independently for each subject, electrode, and frequency, and the P-values were adjusted using the false discovery rate (FDR) of Benjamini and Hochberg (1995). The FDR method was computed using the function *fdr_bh* function from the Mass Univariate ERP Toolbox (Groppe et al. 2011). Furthermore, the Spearman partial correlation of amplitude and duration of the oscillatory bursts, controlling for the peak amplitude of the amplitude envelope time derivative, was computed. Given that the partial correlation presented better correlations than the correlation of peak amplitude vs. duration, the two following linear models were fitted to predict the duration of the oscillatory burst from the amplitude envelope, and the best model was selected through the Akaike Information Criterion (AIC) (Akaike 1974), which weights the goodness of fit taken in account the number of estimated parameters, two for model 1 and three for model 2.

Model 1: OBD = b0 + (b1 * OBEPA).

Model 2: OBD = b0 + (b1 * OBEPA) + [(b2 * PA(dOBEA/dt)].

OBD: Oscillatory Burst Duration.

OBEPA: Oscillatory Burst Envelope Peak Amplitude.

PA(dOBEA/dt) = Peak Amplitude of the Amplitude Envelope Time Derivative of the Oscillatory Bursts.

The AIC of model 1 was subtracted from the AIC of model 2. Negative values of this difference would indicate the best goodness of fit of model 2 concerning model 1.

### Power spectral density

The Power Spectral Density (PSD) of the EEG envelope obtained from the Hilbert transform was obtained independently for the envelope of each of the frequency ranges considered. PSD was obtained using the Matlab function *plomb.* The obtained PSD in the 1–3 Hz range were averaged across subjects and electrodes. The PSD of different frequency ranges between 4 and 6 Hz and 97 and 99 Hz were obtained independently and then averaged across subjects, electrodes, and frequency ranges. The independent computation of PSD in the 1–3 Hz and 4–99 Hz frequency ranges was motivated by the different bursts oscillation duration obtained in these frequency ranges.

### Sanity Check of EEG reconstruction and filters

**Fig. 3:**
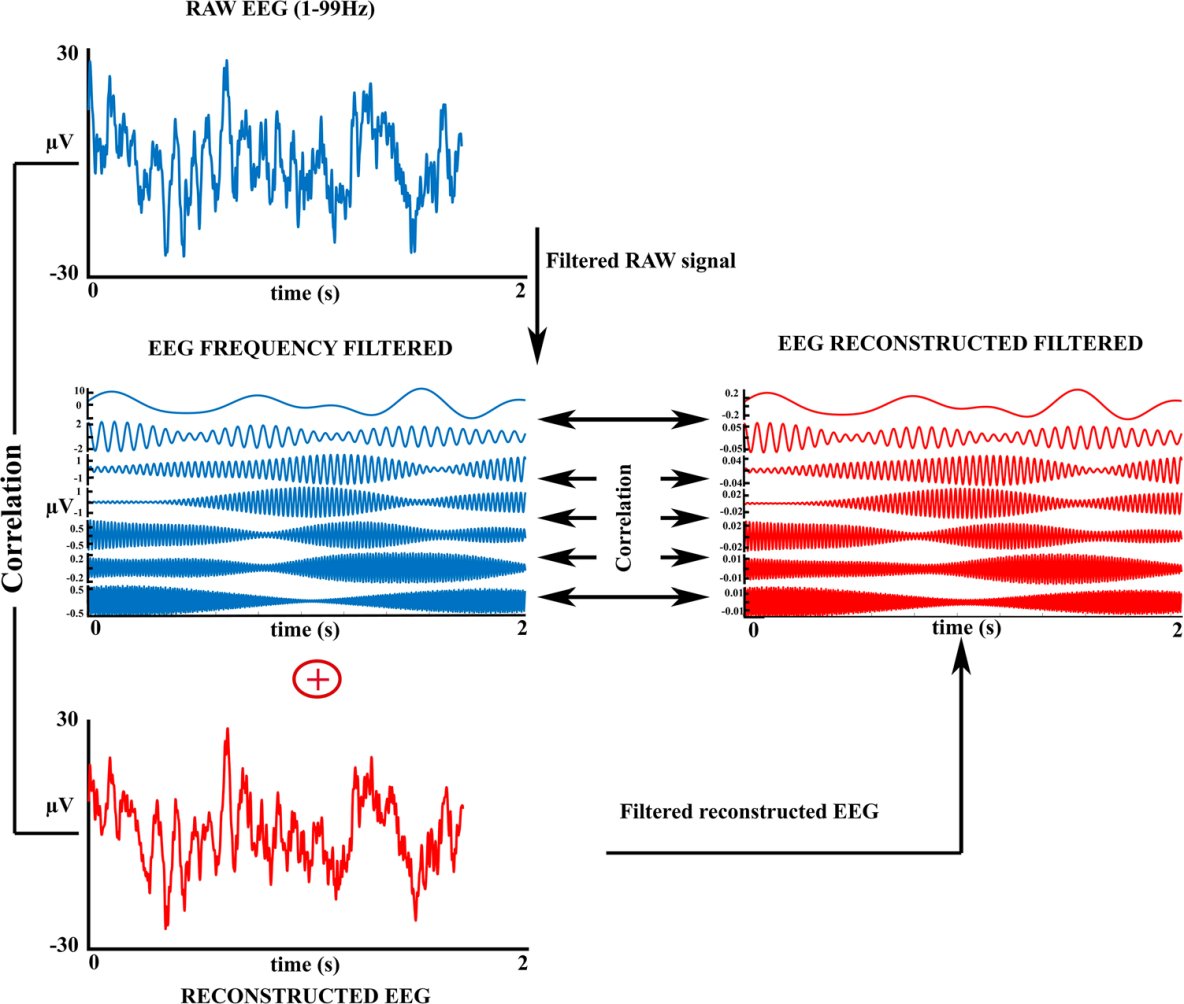
Filters operation checking. The original EEG (above) is correlated with the reconstructed EEG obtained from the algebraic sum of the bank of filtered signals obtained by the filtering process (left side). The filtered signals obtained from the raw EEG are correlated with the corresponding filtered signals obtained from filtering the reconstructed EEG (right side)

Making an effort to show that the used filters are operating are expected, we have reconstructed the EEG as the algebraic sum of all the 33 frequency ranges in which the EEG was filtered (Fig. 3, left). The reconstructed EEG was modulated in amplitude with the slope and intercept of the regression between the reconstructed EEG and the raw EEG. If amplitude artifacts are included in the filtered EEG a gross difference should be obtained between the reconstructed EEG and the original EEG. Rather than using the original EEG, the reconstructed EEG would be compared with the bandpass filtered EEG between 1 and 99 Hz, which corresponds to the frequency range of the 32 frequency ranges used for obtaining the narrow frequency EEG bands. The Spearman correlation of the reconstructed EEG from narrow band filtered signals and the 1–99 band-pass filtered EEG would permit checking the reliability of the filtering procedure to assign power to the filtered EEG signal (Fig. 3, left side).

Furthermore, and in order to check that filters operated correctly, given the high number of artifacts that can arise when misused (Cheveigné and Nelken, 2019), the reconstructed EEG from the filtered signal was filtered with the filters used with the original recorded EEG. Then the correlation between the filtered signals from reconstructed EEG (in which the amplitude modulated sinusoidal signals conforming to the reconstructed EEG are known) and the filtered original EEG recorded signals were obtained for all electrodes and frequency ranges (Fig. 3, right side). If filters operate correctly the correlations should be very high. A total of 960 correlations are obtained by subject (30 electrodes for 32 frequency ranges).

## Results

Figure 2a shows the filtered EEG in different frequency ranges with the overlapping amplitude envelope obtained by applying the Hilbert transform. The visual observation of the filtered EEG supported the presence of continuous oscillatory activity. Oscillatory bursts show a highly variable amplitude and duration, suggesting the possibility of a macroscopic continuous oscillatory activity modulated in amplitude. Figure 2b–e show the detection of troughs delimitating oscillatory bursts using different values for the number of cycles and prominence. The selection of the prominence parameter 0.5*SD seemed to be critical to avoid small-amplitude rebounds in the envelope to be defined as independent oscillatory bursts. The need for 3 cycles to define an oscillatory burst is more motivated by a theoretical criterion of not confusing phasic signals with oscillatory signals.

**Fig. 4:**
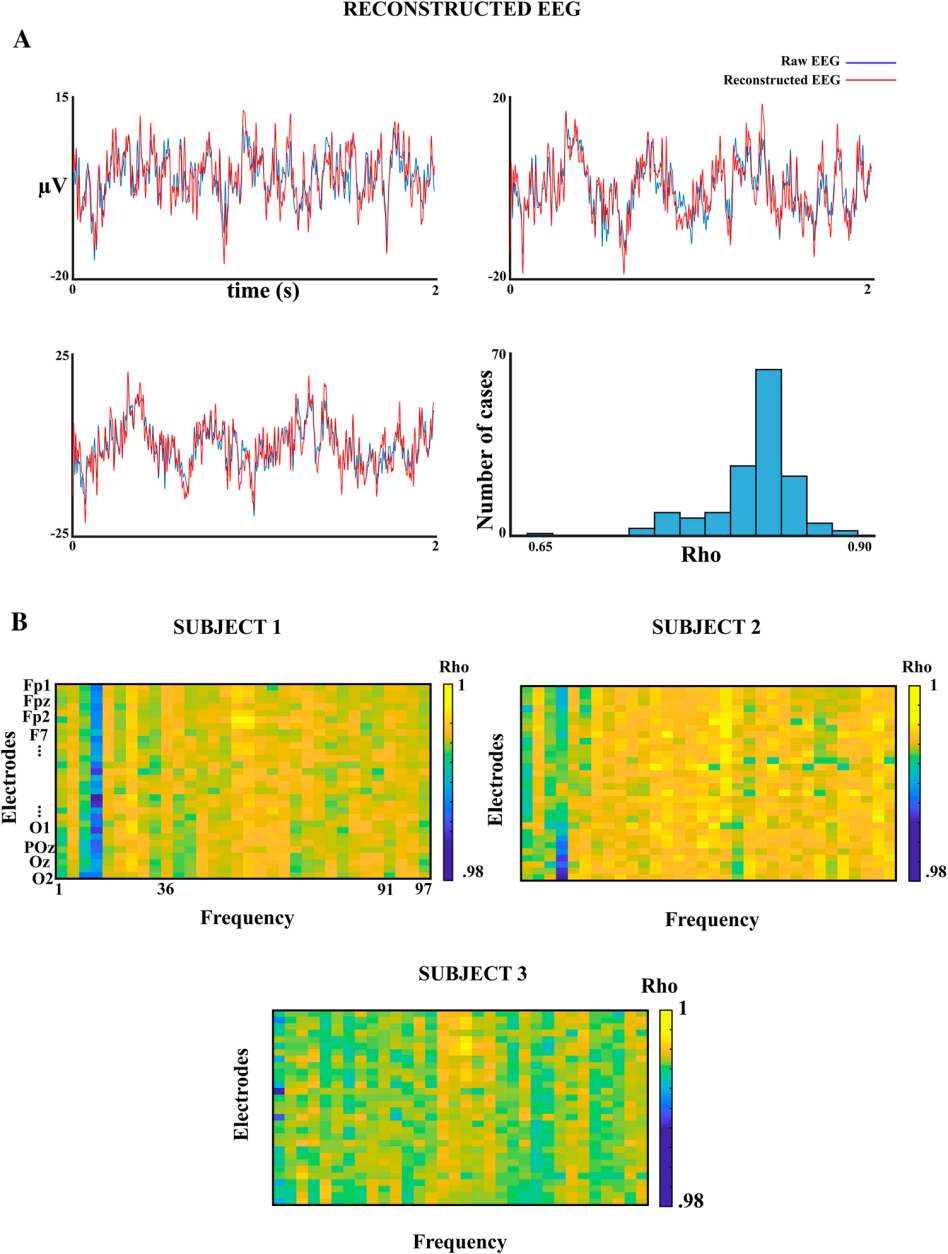
Filters operation checking results. **a** EEG reconstruction from filtered EEG. The figure shows three examples of reconstruction of the EEG from the algebraic sum of the filtered signal in frequency ranges from 1–3 Hz to 97–99 Hz. These reconstructions show a good fitting between the raw signal (filtered in the bandpass 1-99 Hz) and the reconstructed signal. In 4A (below on the right side), the figure displays the histogram of the Spearman correlation between the raw and reconstructed signal for the 30 electrodes and 5 subjects. Please notice the high values of Spearman correlation coefficients. **b** Correlation matrices of filtered signals from reconstructed EEG vs. the signals obtained from filtering the original EEG. The correlations are computed for the 30 electrodes and the 32 frequency ranges considered for three subjects. Frequencies 49–51 were eliminated from all matrices to avoid 50 Hz AC electrical noise

Figure 4a shows a few examples of reconstructed EEG from the algebraic sum of the EEG filtered signals and of the raw EEG. Figure 4a (lower right) shows the histograms of the Spearman correlation coefficients between the reconstructed EEG and the raw EEG for all the subjects and electrodes collapsed. Most correlation coefficients showed a high correspondence between reconstructed and raw EEG. Figure 4b shows the Spearman correlation matrix of the EEG filtered signal vs. reconstructed EEG filtered signal for three subjects. The correlation between the filtered signals obtained from the reconstructed EEG and the filtered signals obtained from the original EEG were in the range 0.98–1 (procedure described in Fig. 3b). Therefore, the operation of the used filters could be considered satisfactory for the purposes of the present report.

**Fig. 5:**
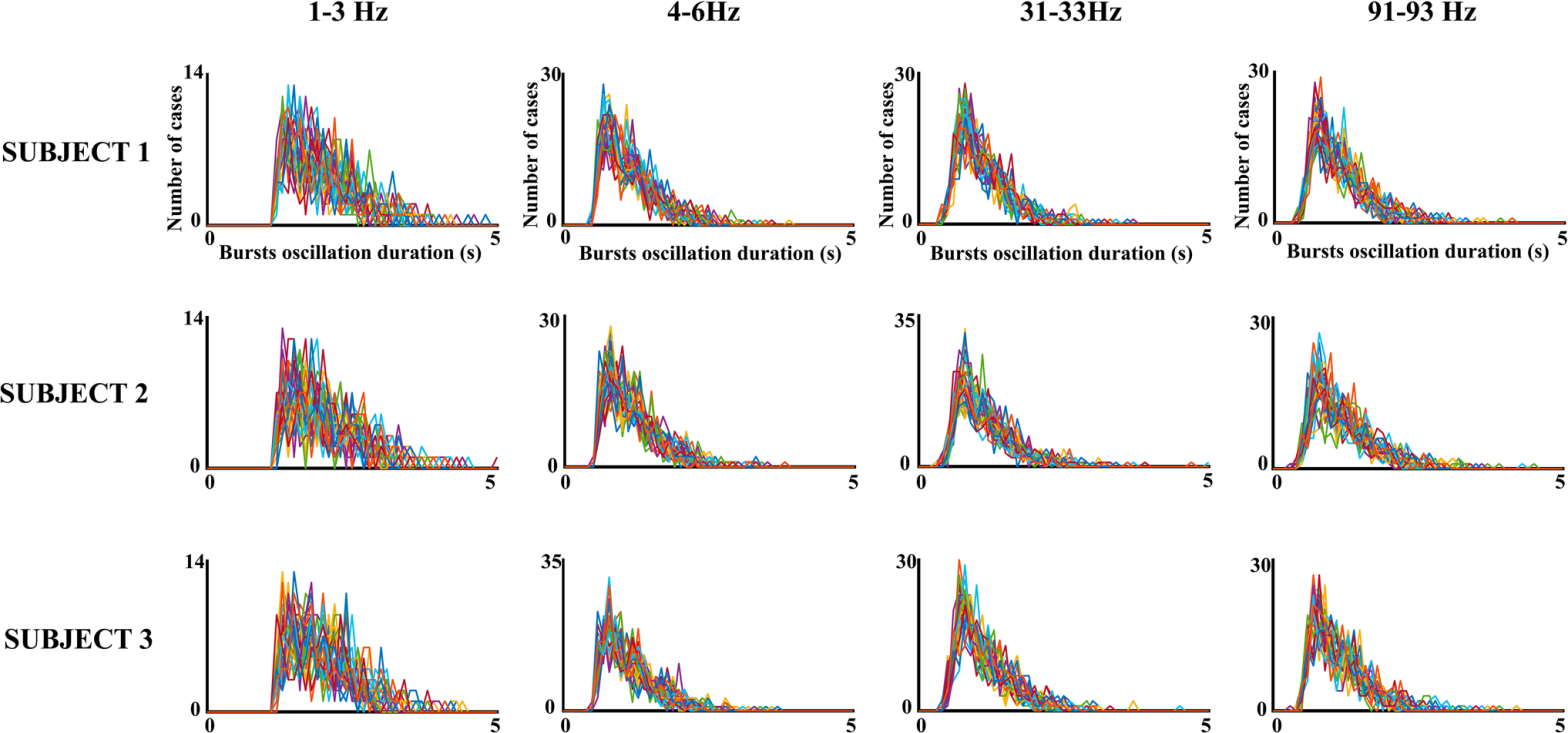
Histograms of oscillatory bursts duration. The figure shows the histograms of the duration of oscillatory bursts during the 3 min’ recordings in different subjects and frequency ranges. The histograms for the 30 electrodes are overlying in the same figure. Notice that oscillatory burst durations are in the range of a few seconds

**Fig. 6:**
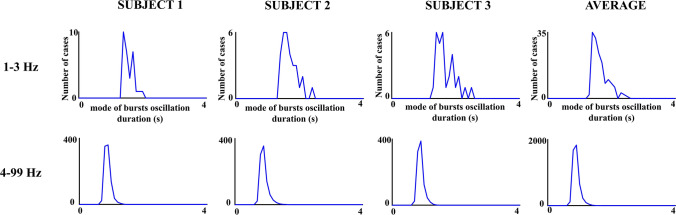
Histograms of the modes of the oscillatory bursts duration histograms. These histograms are displayed for three subjects and the average of all subjects. Please notice the high homogeneity of oscillatory burst mode durations in the collapse of the modes obtained in the different frequency ranges between 4 and 99 Hz. The modes for the frequency range of 1–3.99 Hz presented higher values

Figure 5 shows the histograms of bursts oscillation duration in three subjects for different frequencies. The duration of the oscillatory bursts was in the range of hundreds of ms to a few seconds. Figure 6 shows the histograms of the modes of the burst oscillations duration histograms for different frequency ranges in three subjects and the averaging of the five subjects. The results confirmed a very narrow distribution of the burst oscillation duration modes for the frequency ranges between 4 and 99 Hz, with most modes distributed in the interval of 0.8 s and 0.9 s. For the frequency range of 1-3 Hz, the histograms of the modes of the oscillatory burst durations showed longer durations, with most values being in the interval 1.4 s to 1.5 s. The latter results are clearer when the averaging across subjects, frequencies, and electrodes of the duration histogram modes are observed (Fig. 6, right side). The ANOVA of the bursts oscillation mean duration (within subject factors: frequency ranges and electrodes) showed that the mean duration of 1–3 Hz bursts was longer than the mean durations of 4-99 Hz bursts (F[1,4] = 26,546; p < 0.001; mean duration_1-3 Hz_ = 2 s, SD = 0.022; mean duration _4-99 Hz_ = 1.13 s, SD = 0.020).

**Fig. 7:**
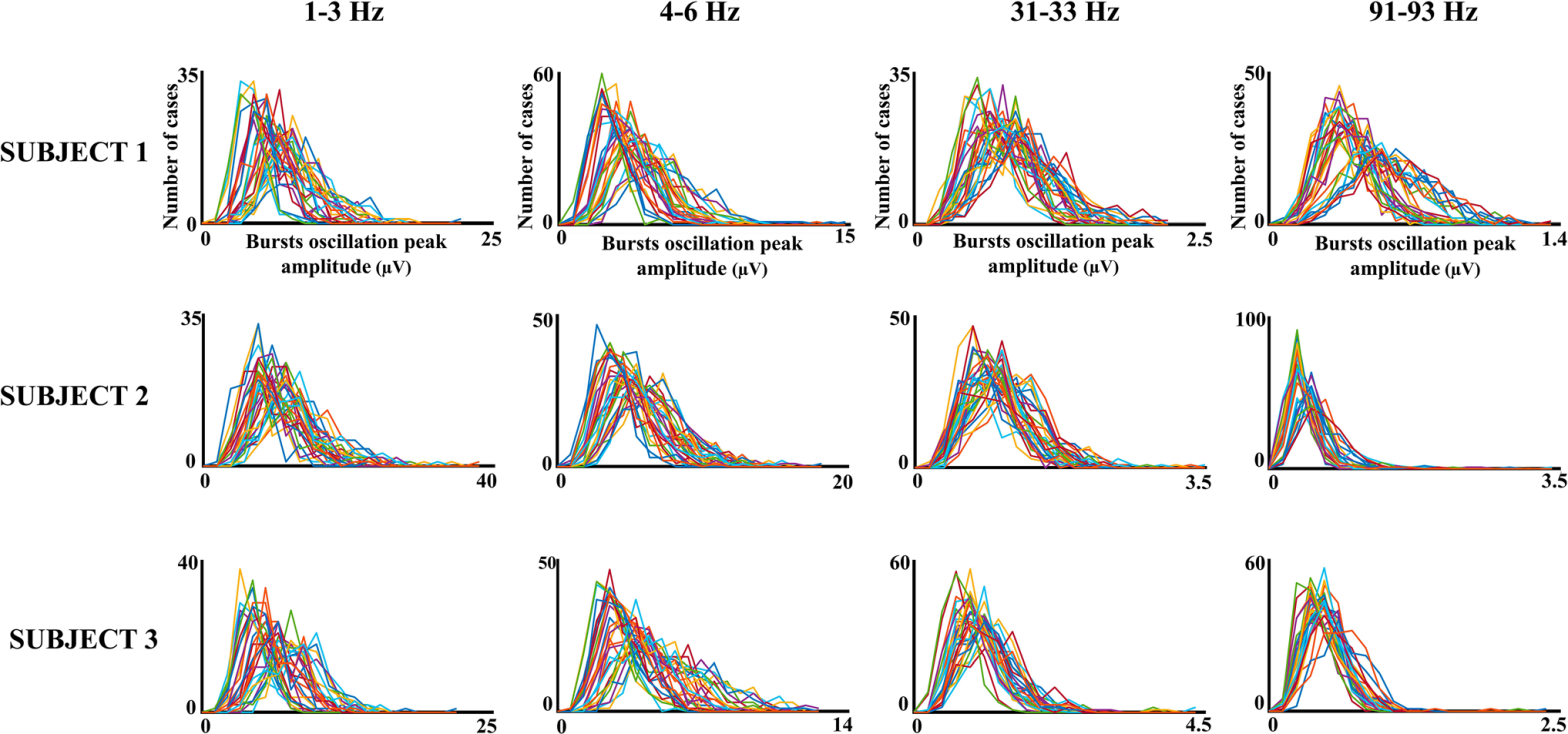
Histograms of oscillatory bursts peak amplitudes. The figure shows the histograms of peak amplitudes of oscillatory bursts during the 3 min’ recordings in different subjects and frequency ranges. The histograms for the 30 electrodes are overlying in the same figure

**Fig. 8:**
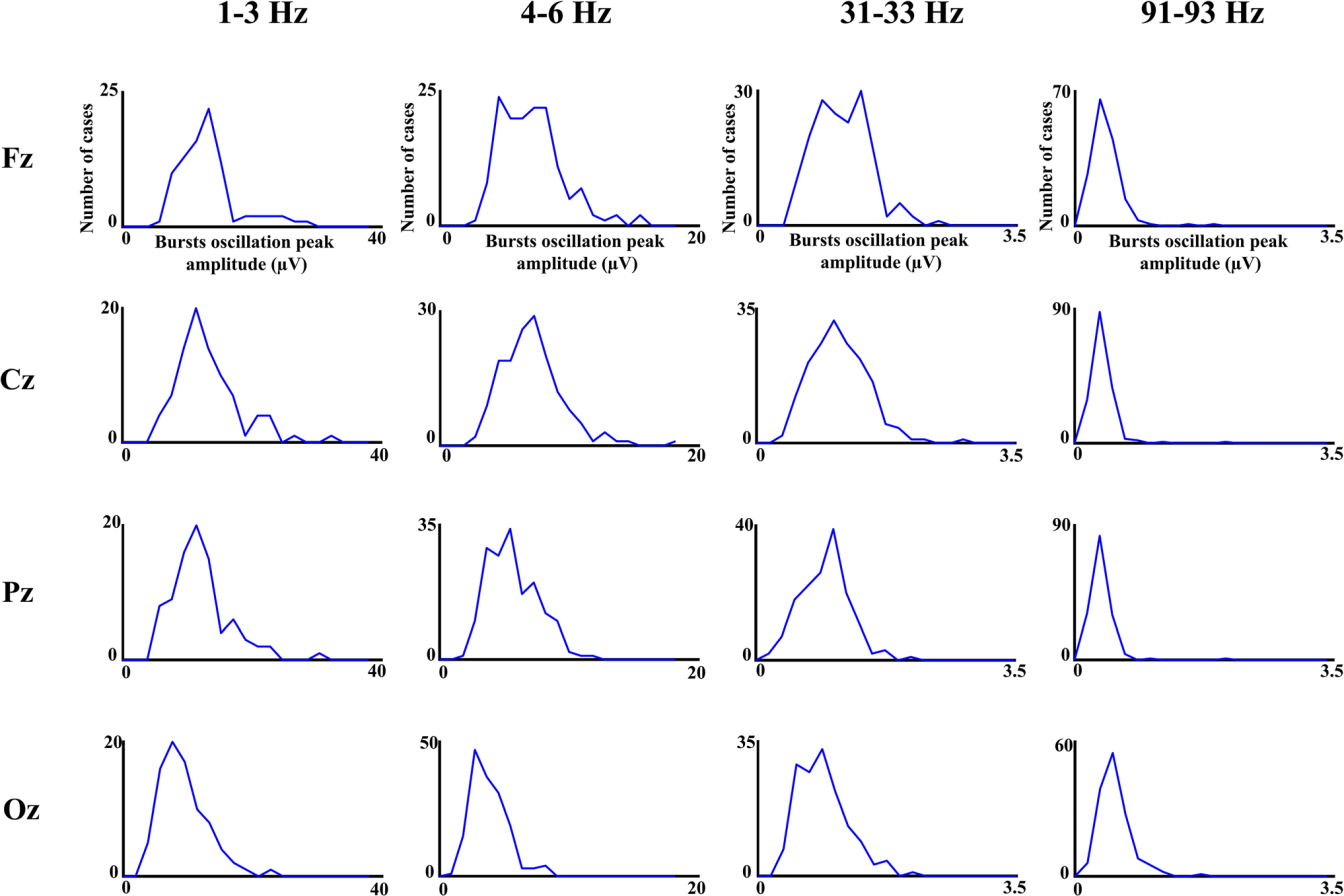
Histograms of oscillatory bursts peak amplitudes details. The histograms of peak amplitudes for the frequencies and electrodes indicated in the figure are represented for a single subject. Please notice the unimodal character of the histograms

Figure 7 shows the histograms of the peak amplitude of the oscillation bursts envelope with the histograms for the different electrodes overlaid. The peak amplitudes were not so homogenous as durations and reflected a high variability in amplitudes across electrodes and frequencies, with a clear decrease of amplitude as frequency increases. To show that the histograms of peak amplitudes were unimodal, the histogram of peak amplitudes in several frequencies and electrodes for a single subject are presented in Fig. 8. The unimodality was common to the five subjects across frequencies and electrodes.

**Fig. 9:**
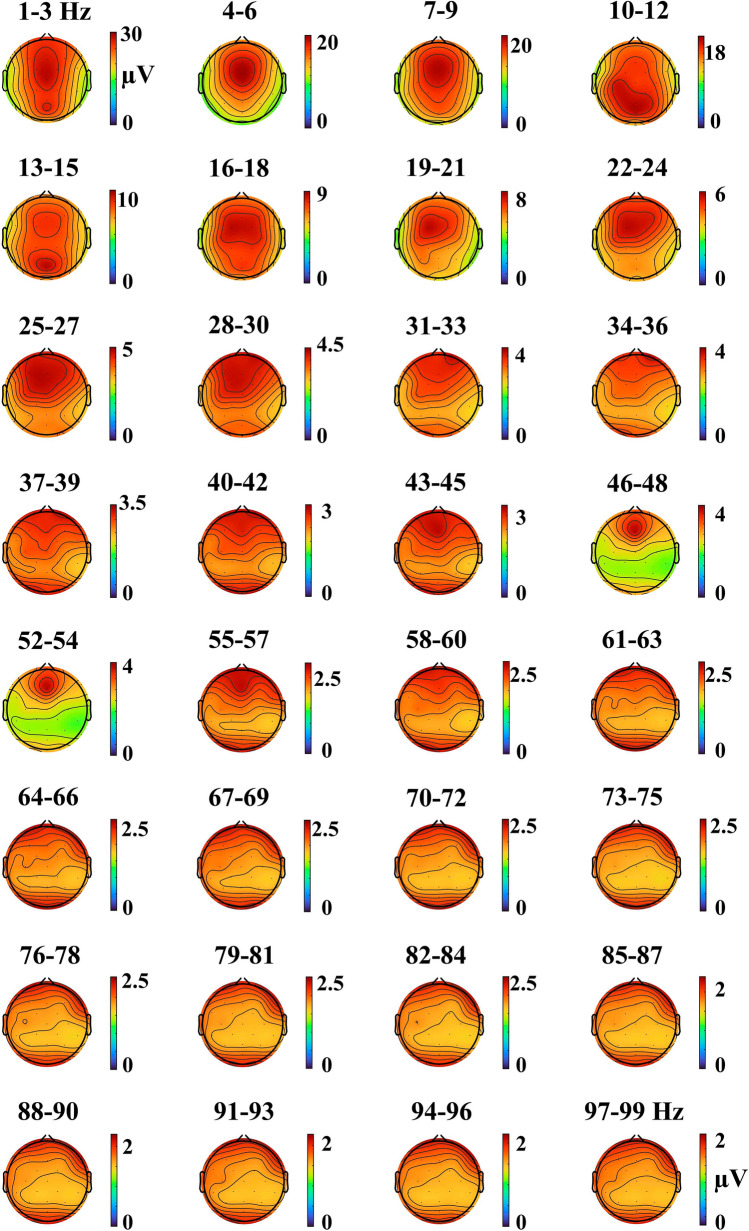
Oscillatory bursts topography. The topographies were obtained as the mean value of the oscillatory bursts envelopes across the 180 s of recorded EEG. The frequencies used for each topography are indicated

Figure 9 shows the topographies of the mean value of the oscillatory burst amplitude envelope across the whole recording time. The delta oscillations (1–3 Hz) showed an anterior–posterior distribution, theta (4–6 Hz) presented a fronto-central topography, alpha (10–12 Hz) was mainly posterior, and beta (16–30 Hz) was frontal; finally, gamma (31–99 Hz) presented a posterior-anterior distribution. Transitional topographies appear around alpha frequency (7–9 Hz and 13–15 Hz).

**Fig. 10:**
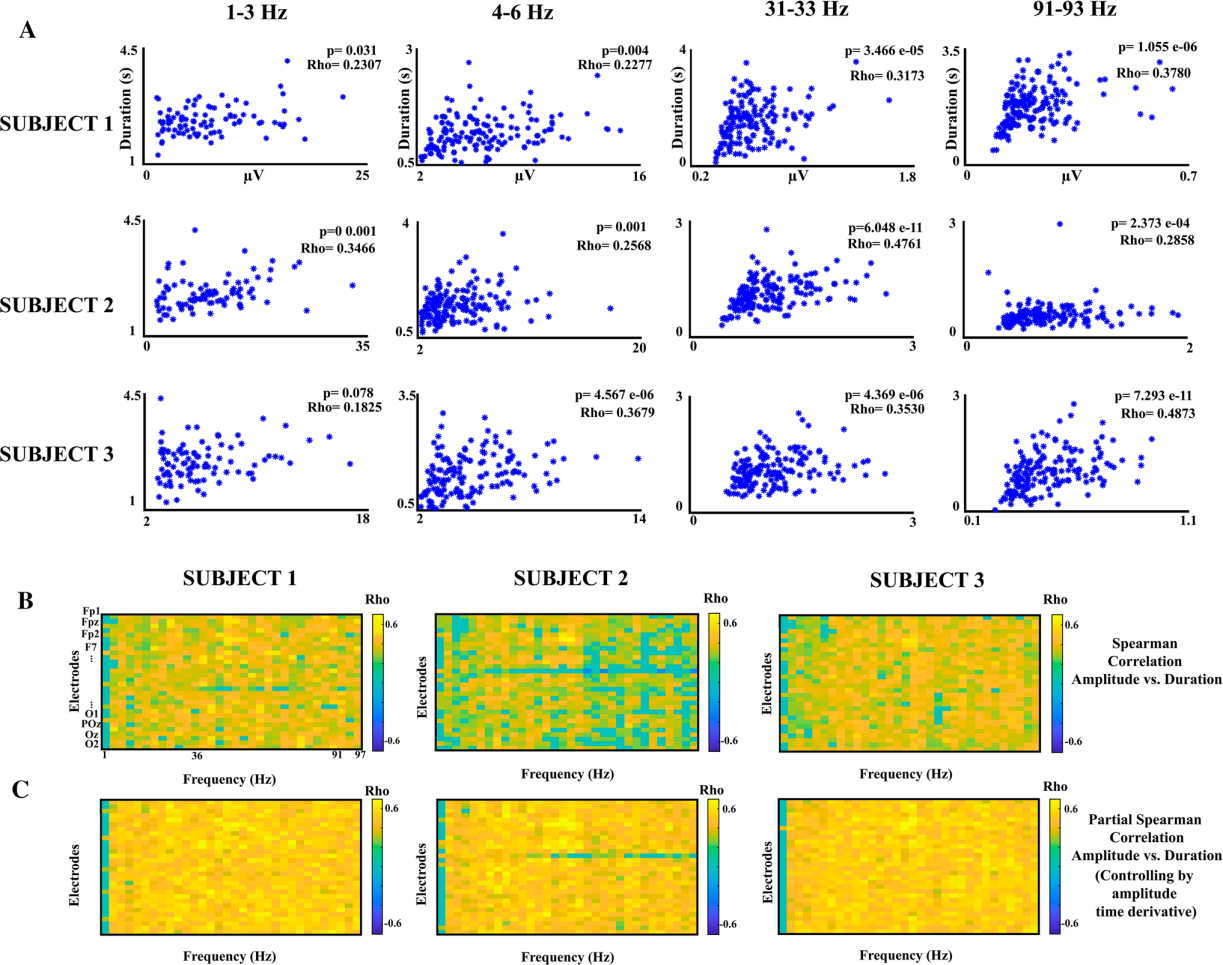
Bursts oscillation duration vs amplitude correlations. **a** The relationship between duration and amplitude for three subjects in four different frequency ranges is represented. **b** Spearman’s correlation matrices of the amplitude vs. duration for three subjects. **c** Spearman’s partial correlation matrices of the amplitude vs. duration (controlling by the peak amplitude of the amplitude envelope time derivative) are represented for three different subjects for all the considered frequency ranges and electrodes. The green color represents those correlations that were not significant after the adjustment by false discovery rate. Frequencies 49–51 have been eliminated from all matrices to avoid 50 Hz AC electrical noise

Figure 10a shows some examples of burst oscillation durations vs peak amplitudes in different frequencies and subjects for the Cz electrode. The graphs show a positive relationship between these two parameters. Figure 10b shows the Spearman correlation coefficient matrices of burst oscillation durations vs. peak amplitudes for three subjects with applied FDR correction. Most of the duration vs. amplitude correlations were significant, although for the low-frequency range of burst oscillations the number of significant correlations was smaller. Figure 10c shows the partial correlation matrices of the peak amplitude vs. duration (controlling by the peak amplitude of the amplitude envelope time derivative). The correlation values were increased for 10B, suggesting that indeed the peak of the amplitude envelope time derivative is also a predictor for the burst oscillation duration. The latter results motivated the statistical comparison of the two models described in the Methods section for predicting the duration of oscillatory bursts. The pattern of correlations for the two subjects not displayed was very similar to those displayed in Fig. 10.

**Fig. 11:**
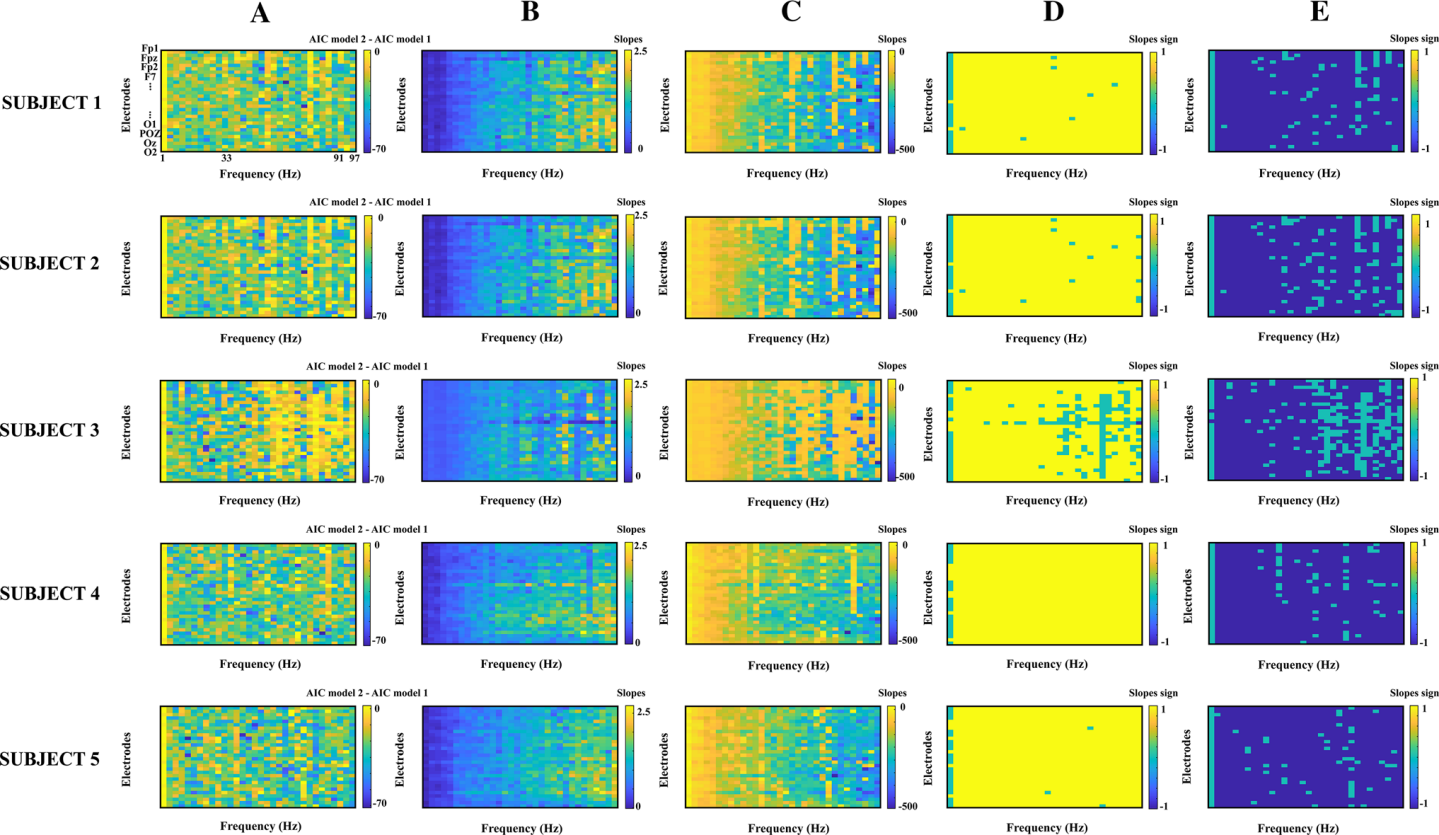
Assessment of model 1 and model 2. **a** Models difference of Akaike Information criteria (AIC model2 – AIC model 1). **b** Slopes of the peak amplitude of the oscillatory burst envelope term in model 2. **c** Slopes of the Peak Amplitude of the envelope amplitude time derivative term in model 2. **d** Signs of the slope matrices displayed in 10B. **e** Signs of the slope matrices displayed in 10C. The non-significant terms after applying the False Discovery Rate method are labeled in green color in **d** and **e**. Frequencies 49–51 have been eliminated from all matrices to avoid 50 Hz AC electrical noise. The results are presented for the five subjects, 30 electrodes, and 32 frequency ranges

Figure 11a shows the AIC in model 1 subtracted from AIC in model 2. For most electrodes and frequencies, the AIC differences indicate that model 2 presented the best goodness of fit than model 1 taking into account the number of estimated parameters. Figure 11b and c show the absolute values of the slopes for the first and second terms of model 2, respectively. Figure 11d and e show the signs of these two slopes, indicating when these terms were non-significant after applying the FDR correction. Most frequencies and electrodes presented a positive slope for the first term of model 2 (peak amplitude of bursts oscillation envelope) and a negative slope for the second term (the peak amplitude of the oscillatory bursts amplitude envelope time derivative). These results suggest some sort of negative feedback for fast-developing oscillations. For the low-frequency components (1–3 Hz) the statistical significance of the terms of model 2 was not significant for a higher number of electrodes than in the 4–99 Hz range.

**Fig. 12:**
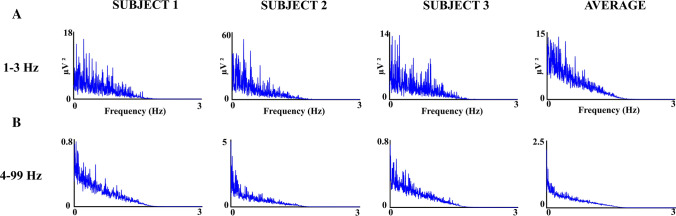
Power spectral density of the oscillatory bursts envelope. Above: The Power Spectral Density (PSD) of the burst oscillations amplitude envelope of the 1–3 Hz range collapsed across electrodes are represented for three subjects. The averaging of the five subjects is displayed in the right part of the figure. Below: the collapse across electrodes of the PSD in the frequency ranges from 4–6 Hz to 97–99 Hz are displayed for three subjects. The averaging across frequencies and electrodes is represented on the right side. Please notice the low frequency content of the oscillatory bursts amplitude envelope, the 1/f decaying of the PSD, and the absence of defined peaks

Figure 12 shows the PSD of the amplitude envelope collapsed across electrodes in three subjects and the average of the five subjects. The envelope of the oscillatory bursts of the frequency range 1–3 Hz was analyzed independently from the rest of the frequency ranges (4–99 Hz). The PSD of the latter range was collapsed across electrodes and frequencies given their more homogenous behavior. In both cases, a decaying 1/f spectral power can be observed with all the power concentrated in the range of 0–2 Hz, indicating the low-frequency oscillatory characteristics of the amplitude envelope of oscillatory activity in the range of 1–99 Hz, and the absence of defined peaks suggesting the absence of a rhythmic oscillatory pattern.

**Fig. 13:**
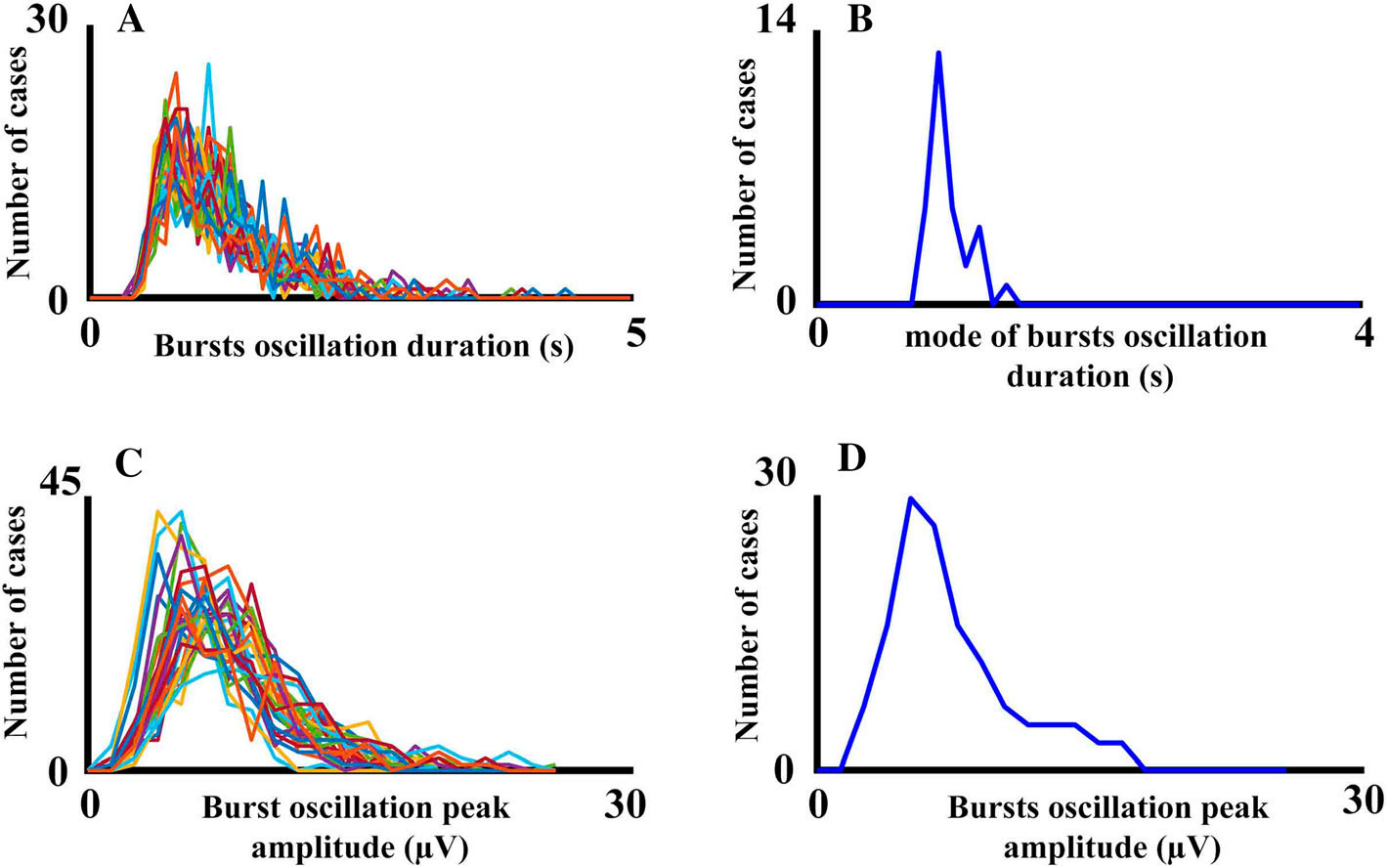
Statistical properties of alpha oscillatory bursts. **a** Histograms of oscillatory bursts duration of the 30 recorded electrodes. **b** Histograms of the modes of the oscillatory bursts duration histograms of all the electrodes. **c** Histograms of oscillatory bursts peak amplitudes of the 30 recorded electrodes. **d** Histograms of oscillatory bursts peak amplitudes for the electrode Oz

Figure 13 shows the statistical properties of the oscillatory bursts in the alpha range (10–12 Hz), showing the same typical characteristics of duration in the range of few seconds as the other considered frequencies (Fig. 13a), modes of duration histograms around 1 s (Fig. 13b), unimodal histograms for peak amplitude of oscillatory bursts (Fig. 13c), and the detail of the peak amplitude histograms for the electrode Oz displaying a broad amplitude distribution and unimodality (Fig. 13d), indicating that peak amplitude of oscillatory bursts in the alpha range is a continuous variable in a broad range of amplitudes. The correlations between duration and peak amplitude of oscillatory bursts, the correlations between duration and peak amplitude of oscillatory bursts (controlling for the peak amplitude of the time derivative of oscillatory bursts amplitude), and the demonstration of the best fitting of model 2 with respect to model 1 are displayed for the alpha rhythm in the fourth column of Figs. 10b–c and 11a–e, respectively. All these results for the alpha frequency are quite similar to those obtained for the other analyzed frequencies in the range 4–99 Hz.

## Discussion

The present report confirms the presence of oscillatory bursts with durations from hundreds of milliseconds to a few seconds in frequencies ranging from 1 to 99 Hz, with 0.8–0.9 s as the most frequent duration for oscillatory bursts. The amplitude envelope of the oscillations showed peaks amplitude with unimodal distributions suggesting a continuous activation level in the networks generating the macroscopic oscillatory bursts. Peak Amplitude and duration of oscillatory bursts presented positive correlations that were increased when controlling by the peak amplitude of the time derivative of the amplitude envelope. Then, a linear model to predict the duration of the oscillatory burst by the amplitude and by the time derivative of amplitude was confirmed. The latter results suggested that the duration of oscillatory bursts as recorded in the extracranial EEG are negatively modulated by the envelope amplitude time derivative, limiting the duration of oscillatory bursts rapidly developing. All these results were present, although less clear for low-frequency oscillations (1–3 Hz). The power of the amplitude envelope of oscillatory bursts was concentrated in a very narrow low frequency around 0–2 Hz. The present results suggest that a structured modulation of the amplitude of oscillatory burst occurs in the neural networks supporting human EEG.

The present analysis showed the presence of amplitude modulation of oscillatory activity in the macroscopic human EEG in a broad frequency range with durations relatively long, in the range of hundreds of milliseconds up to a few seconds. This result is compatible with the view of continuous oscillatory activity modulated by amplitude, expressed by van Ede et al. (2018), in one of the possible scenarios underlying the oscillatory bursts generator. This possibility is different from the idea of short-lived bursts of oscillatory activity which has been shown in different experimental intracranial recording settings. For instance, the conclusion that beta oscillations of long duration are a by-product of single-trial averaging rather than a genuine long-duration beta oscillatory burst has been drawn. Beta bursts which are short-lived would produce by averaging across trials long-duration beta burst (Feingold et al. 2015). Sherman et al. (2016) and Shin et al. (2017) have shown that the beta oscillations of long durations are due to averaging of short-lived beta bursts, in both mouse and human MEG. Similarly, during Working Memory tasks non-periodic gamma bursts of around 100 ms occur during the encoding and decoding phases of WM tasks, and only trial averaging produces the impression of long durations of gamma bursts (Lundqvist et al. 2016). This controversy can be overcome if the macroscopic continuous oscillatory activity modulated in amplitude, in the duration range of few seconds, as obtained in the present report, is considered as the algebraic sum of a myriad of microscopic short-lived microscopic events in the range of the hundreds of milliseconds when not very strict amplitude criteria are used to define an oscillatory burst. However, if this is the case, the highly structured pattern of macroscopic oscillatory activities obtained in the present report would imply that microscopic neural oscillations must be chained somehow to produce the organized pattern observed at the macroscopic scale. The obtained topographies support that the energy assigned by the Fourier analysis to each frequency range corresponds to the traditional brain rhythms, given that the spatial distribution of the envelopes of the oscillatory bursts is quite similar to previous results for brain rhythms topographies (Niedermeyer and Lopes da Silva 1999; Rodríguez-Martínez et al. 2017). But also, that the obtained oscillatory burst are genuine oscillations and not methodological artifacts.

The unimodal distribution of peak amplitudes suggests that there are not very defined thresholds for generating an oscillatory burst, at least from the macroscopic point of view. If a fixed threshold is needed to generate an oscillatory burst a bimodal distribution should be expected in peak amplitude distributions, one corresponding to subthreshold and another corresponding to suprathreshold oscillations that would generate a cooperative phenomenon somehow similar to an action potential, but at the network dynamics level. However, as indicated before, it is always possible that microscopic networks could have a defined threshold to fire an oscillatory burst, but at the macroscopic averaging it could be perceived as a continuous of peak amplitude values.

A significant relationship is found for the amplitude vs. duration relationship for most electrodes and frequencies. This relationship is also described by Feingold et al. (2015) for beta bursts. The underlying explanation would be in a limited capacity of recurrent networks to increase the level of activity, and then oscillatory bursts to achieve high amplitude would need long developing times. An interesting point obtained from histogram durations is that the mode of oscillatory burst durations was very constant for all electrodes and frequencies, around 0.8–0.9 s for most frequencies, and a bit longer (1.4 s) for the 1–3 Hz range. This result suggests relatively fixed internal dynamics for oscillatory burst generators, and particularly for the ignition of inhibitory networks that would limit the duration of oscillatory bursts. As indicated before, this result is not contradictory at a macroscopic scale with much shorter durations of oscillatory bursts at a microscopic scale (Feingold et al. 2015; Lundqvist et al. 2016).

A positive feedback loop can be proposed for the increase in the amplitude of oscillatory bursts, while negative feedback would explain the decaying phase. These two phases are represented in model 2 in which the duration of the oscillatory bursts is predicted by a combination of a positive peak amplitude term and a negative term of the peak amplitude of the amplitude envelope time derivative. The first term would be embedded in an excitatory recurrent network, while the second term would be embedded in an inhibitory recurrent network, both with temporal dynamics in the range of few seconds. Fast developing oscillations would generate high negative feedback loops to limit the duration of the developing oscillatory bursts. Therefore, rapid changes in the amplitude of oscillatory bursts would increase dramatically the activity of inhibitory networks limiting the activity of excitatory projections. The excitatory-inhibitory mechanism proposed for the amplitude envelope modulation would be completely similar to that proposed for the generation of oscillatory activity based on a combination of excitatory AMPA and inhibitory GABA activity (reviewed in Jensen et al. 2019). In the so-called pyramidal-interneuronal network gamma model (PING) for generation of gamma and beta rhythms, recurrent inhibitory GABA activity from interneurons would shape the oscillatory activity in local circuits, while pyramidal cells would excite them through AMPA receptors (Whittington et al. 2000; Tiesinga and Sejnowski 2009). For slower frequency rhythms long distance excitatory-inhibitory septo-hippocampal (Theta rhythm) and thalamo-cortical (alpha rhythm) interactions are needed. The oscillatory bursts behavior obtained in the present report suggests that a pattern of excitatory-inhibitory activity must be sculpturing the oscillatory bursts of different frequencies. Therefore, the activation-inhibition mechanism would be operating at different time scales. In the range of a few hundred or tens milliseconds for generating the different brain rhythms, and in the scale of 1 or few seconds for the amplitude modulation of brain rhythms.

One striking characteristic of the present analysis is the different results obtained by oscillations in the delta range concerning those in the frequency ranges from theta to gamma for a variety of parameters: the mode of histograms of oscillatory bursts duration and a lower statistical significance for the modeling of oscillatory durations by amplitude and the time derivative of envelope amplitude. The longer duration of oscillatory bursts in the low-frequency range is completely justified by the methodological limitation imposed of a minimum number of cycles to consider the extraction of a genuine oscillatory burst. But the lower statistical significance of the duration prediction by amplitude and its time derivative should be due to some particular dynamics of these oscillatory frequencies, which may be linked to the longer duration of oscillatory bursts making that activation and inhibition loops would have a lower possibility to interact to control the network activity.

The Low-frequency EEG oscillations (< 0.5 Hz) and the gamma power have been related to the low-frequency oscillations of functional magnetic resonance during (fMRI) resting-state (He et al. 2008). De Pasquale et al. (2010) proposed that the best correlations of power of the magnetoencephalographic signal at the source level with fMRI during resting state occurred at theta, alpha, and beta bands. The low frequency of the oscillation envelopes, in the range of 0–2 Hz, but concentrating most of the energy in the very-low-frequency range, as described in the present report, supports the concept that at least part of the low-frequency oscillations of fMRI during resting state would be related to the amplitude modulation of frequency oscillations in a broad frequency range. The correlation of fMRI and EEG envelopes of broad frequency bands should be computed to test such a possible relationship. On the other hand, the absence of defined peaks in the PSD of the envelope amplitudes suggests that oscillatory bursts are relatively random and do not present a defined periodicity during resting state. The latter results are in agreement with the obtained fano factor higher or equal to one obtained for inter-event beta intervals (Shin et al. 2017), although other authors have reported a fano factor lower than 1 suggesting a more rhythmical behavior for oscillatory events (Neymotin et al. 2020).

From a functional point of view, the presence of envelopes for oscillatory activity in the range of seconds would permit to establish a temporal framework with enough duration to permit the processing of cognitive tasks. In this sense, Pöppel (Schleidt et al. 1987) initially based on transcultural ethological studies of the duration of purposive acts, has proposed a more general framework in which cognitive processes occur in a temporal extent of a few seconds. Extensive evidence in the duration of perceptual, motor, and cognitive processes tends to support such a conclusion (Pöppel 1987; Gómez et al. 1995). However, no compelling physiological evidence has been proposed to support such a claim. The presence of oscillatory envelopes in the range of few seconds obtained in the present report would support the idea of neurophysiological integrative processes in such a temporal range.

Two possible limitations appear in the present report: (i) As the methodological constraint imposed of very long recording periods without artifact would potentially bias the selection of subjects to high aroused individuals, the present results should be replicated controlling for situational stress and in a broader subjects sample; and (ii), the EEG has been described as the combination of periodic and aperiodic components (broadband offset and exponent) (Donoghue et al. 2020). The present report did not consider these different components. Therefore, the amplitude and duration parameters of oscillatory bursts would be modified by these aperiodic components in the present analysis. A complete statistical description of the oscillatory bursts behavior would require a trial-by-trial extraction of the aperiodic components. As the present analysis has been computed on spontaneous EEG data, the periodic and aperiodic would sum up giving a macroscopic picture of oscillatory bursts independent of the possible different origins of amplitude modulation.

## Conclusions

The present report shows a continuous oscillatory activity modulated by amplitude in a broad frequency range, including the alpha rhythm. The temporal span of the amplitude modulation is in the order of hundreds of milliseconds to a few seconds compatible with previous ideas of this temporal framework for integrative motor, sensory and cognitive processes. The results are compatible with a continuous modulation of the amplitude of oscillatory activity showing negative feedback for rapidly developing oscillatory bursts.
